# An Educational Workshop to Improve Neurology Resident Understanding of Burnout, Substance Abuse, and Mood Disorders

**DOI:** 10.15766/mep_2374-8265.11164

**Published:** 2021-07-01

**Authors:** Ryan Donaghy, Shiori Tomatsu, Patrick Kerns, Courtney White, Jeffrey Ratliff

**Affiliations:** 1 Resident, Department of Neurology, Northwestern University; 2 Medical Student, Sidney Kimmel Medical College at Thomas Jefferson University; 3 Fellow, Department of Neurology, University of Virginia; 4 Fellow, Department of Neurology, Thomas Jefferson University; 5 Clinical Assistant Professor, Department of Neurology, Thomas Jefferson University

**Keywords:** Burnout, Role-Play, Wellness, Substance Abuse, Physician Impairment, Neurology, Well-Being/Mental Health

## Abstract

**Introduction:**

Burnout, substance abuse, and mood disorders are prevalent among neurology residents. Increased recognition of concerning behaviors might encourage more access to mental health resources and reduce burnout.

**Methods:**

We created an educational resource reviewing burnout, substance abuse, and mood disorders for neurology residents. This resource included an online module (control) and a role-play scenario offered only to one cohort (intervention). Online surveys assessed knowledge as well as confidence in the ability to recognize concerning behaviors. A practical assessment using a previously published “Stressed Resident” video was also conducted among resident cohorts.

**Results:**

Of neurology residents, 18 participated in the activity, with nine in the control group and nine in the intervention group. In the postvideo survey, the residents who participated in a role-play activity outperformed a control cohort of their peers when identifying signs of burnout, mood disorders, and substance abuse portrayed in the video (84% vs. 72%; *t* test, *p* = .01). Residents indicated increased confidence in the ability to recognize symptoms of maladaptive stress as well as identify resources for themselves and peers. Participants demonstrated no difference in knowledge-based questions scores on pre- and postactivity assessments.

**Discussion:**

Our educational resource improved resident ability to recognize signs of maladaptive stress and to identify residents that are a risk to patient safety. The activity is easy to implement and can be easily adapted outside neurology. Limited sample sizes may limit the ability to demonstrate this tool's impact on knowledge of burnout, substance abuse, and mood disorders.

## Educational Objectives

By the end of this activity, learners will be able to:

1. Define symptoms of burnout, substance abuse, and mood disorders, particularly in resident physicians.
2. Identify symptoms of burnout, substance abuse, and mood disorders in structured role-play activities.
3. Recognize the behaviors associated with maladaptive stress in a peer resident.

## Introduction

Burnout is a mental state consisting of emotional exhaustion, depersonalization, and a decreased sense of personal accomplishment,^1^ often manifesting as a reaction to occupational stress.^2^ Physicians experiencing burnout are more likely to experience job dissatisfaction^3,4^ and self-report an increased rate of perceived medical errors.^4,5^ Physicians experience nearly twofold higher risk of burnout compared with the general population.^6^ The burnout syndrome is delicately linked to the efficacy of the health care system, and increasing rates of burnout threaten the provision of medical care via increased rates of patient dissatisfaction, longer patient recovery times, lower-quality patient care practices and attitudes, and higher rates of early physician retirement.^4,7–9^

Physicians in residency training report high rates of burnout symptoms, with up to 45% describing at least one symptom of burnout irrespective of training program specialty.^10^ When burnout rates in residents are analyzed by training focus, subspecialties including general surgery, emergency medicine, and neurology all show a higher rate of burnout compared to colleagues in internal medicine. A reported 70% of neurology trainees experience at least one symptom of burnout.^10,11^ These risks remain high throughout a neurology career, as 60% of practicing neurologists in the United States continue to report at least one symptom of burnout.^12^ Neurology resident training is therefore a target for enabling residents with the ability to identify signs of burnout in both themselves and others.

Related to burnout, residency training is also a time when substance abuse and depression can intensify. Burnout in residents has been shown to increase the risk of self-perceived medical errors, which correlates with developing depression and a reduction in quality of life.^5^ In a pooled meta-analysis, up to 30% of residents met criteria for depression or experienced depressive symptoms.^13^ Internal medicine residents meeting criteria for burnout are more likely to screen positively for depression.^9^ Substance abuse in residency further devitalizes the training experience, and residents report high rates of alcohol abuse and binge drinking.^14,15^ Concerning rates of alcohol abuse and dependency continue to afflict postresidency physicians across specialty disciplines.^16^ Depression, substance abuse, and suicidality share overlapping characteristics, and physicians die by suicide at rates higher than their peers in the general population.^17^ Strategies to reduce rates of depression and substance abuse in residency are paramount in the emerging physician suicide crisis.

The ACGME stipulates in the Common Program Requirements that trainees in a residency program must “demonstrate an understanding of their role in … recognition of impairment, including from illness, fatigue, and substance use, in themselves, their peers, and other members of the health care team.”^18^ In addition to this, requirements state that a training program must educate “residents in identification of the symptoms of burnout, depression, and substance abuse, including means to assist those who experience these conditions. Residents and faculty members must also be educated to recognize those symptoms in themselves and how to seek appropriate care.”^18^ Educational initiatives to facilitate resident physicians’ recognition of psychological impairment in themselves and co-residents go beyond the satisfaction of accreditation requirements. Prior studies have illustrated that resident physicians may lack insight into maladaptive responses to stress.^19^ Residents score worse than attending cohorts in recognizing simulated maladaptive responses to occupational stress.^19^ Thus, curricula to help increase this insight have been sought. Curricula available in *MedEdPORTAL* have focused on activities that build resilience through group reflection,^20^ use didactic teaching to build resilience skills,^21^ or use peer mentorship to build resilience among junior trainees.^22^ Rather than build resilience, we present an activity that uses online modules and role-play to help residents learn about the topic of maladaptive stress response, and also to build the skill of recognizing its associated behaviors. Where prior curricula have taught the topic of burnout, we build on these resources to use role-play as a means of imbuing an observational skill. Role-play has been demonstrated to help residents with skills requiring sensitive communication.^23,24^ Assessment of our activity's ability to improve resident skill at recognizing maladaptive responses to occupational stress was also performed. While the value of recognizing burnout in the self is established, we believe that our activity could allow residents to better support one another by recognizing concerning behaviors and intervening with their at-risk peers. We suspect that by increasing the ability of co-residents to recognize concerning behaviors and intervening when they see them, more residents may seek the resources available to them to address their own mental health concerns.

## Methods

### Educational Approach/Overview

We developed a two-part educational activity for the neurology resident learner, with a focus on burnout, substance abuse, and mood disorders in residency training. The educational activity was delivered via an online module and a group role-play activity.

### Participants and Educational Setting

The Thomas Jefferson University neurology residency program, a large academic clinical program, consisted at the time of nine PGY 2 residents, nine PGY 3 residents, and nine PGY 4 residents. In July of the PGY 2 year, residents attended a neurology boot camp to help facilitate the transition from internal medicine preliminary year training to clinical neurology. Educational activities for this 10-day course included education on neurologic emergencies, orientation to clinical services in the department, and education on issues regarding burnout in neurologists. The time frame of our educational activity extended from July 2019 (PGY 2 boot camp) through September 2019 (final assessment).

Access to a personal computer, tablet, or cellphone was required for viewing the online module. For the role-play sessions, a dedicated room with shared tables could be used. For the video scenario viewings, a small room with standard presentation audiovisual setup could be used. The online module and all survey materials are available in the provided appendices.

### Educational Materials

#### The online module

We developed an online module using the Rise 360 course e-learning software from Articulate. To create the online module, we broadly surveyed the available literature on burnout, substance abuse, and mood disorders in medical trainees. The first part of the module focused on substance abuse in residency, and the second part focused on burnout and mood disorder symptoms. The module included presented information on the scale and impact of various forms of maladaptive stress as well as information on behaviors associated with these responses. Informative and interactive elements were both present in the module. Included here as a zip folder (Appendix A), the module took approximately 30 minutes to complete by our testing. We did not collect metadata from our learners on how much time they spent completing the module.

#### Role-play sessions

The role-play session consisted of three vignettes designed as scenarios in which one resident would initiate an empathetic conversation about another resident's scripted struggles with burnout, substance abuse, or a mood disorder. This activity occurred in a large conference room with no additional audiovisual needs. We assigned residents to groups of three, with one resident serving as the struggling resident, another serving as their concerned peer, and the last serving as an independent observer and conversation facilitator. A PGY 4 resident moderator circulated and answered resident questions regarding the activity or vignettes. These senior resident moderators did not undergo any specific training in the administration of this activity besides familiarizing themselves with the format of the activity and the vignettes ahead of time. The scripts and discussion prompts are provided in Appendix B.

#### Educational activity

Contemporaneously with neurology boot camp, we assigned all residents the online module as part of a greater residency program educational initiative regarding the identification of maladaptive responses to stress. The PGY 2 class was assigned to complete the module prior to their participation in the role-play activity. We delivered the module via an emailed hyperlink and hosted it on our institution's Articulate Rise 360 license. While not directly solicited from the residents, no one raised concerns about being able to access the module on their personal computer browsers. The module is available in Appendix A. Our platform did not allow for tracking of module completion.

The role-play sessions occurred in person during a scheduled afternoon of the boot camp curriculum. This educational activity was implemented before COVID-19-related social distancing requirements. We have not since replicated the role-play in the virtual teleconference environment.

### Educational Activity Assessment

We administered multiple assessments to measure the effect of the role-play activity. All resident participation in these assessments was voluntary. The Thomas Jefferson University Institutional Review Board deemed the research exempt.

The PGY 2 and PGY 3 classes were administered a survey (Appendix C) on their confidence and knowledge of the material prior to the availability of the online module. The format of this survey included Likert scales assessing their confidence in topics related to maladaptive responses to stress as well as multiple-choice questions regarding material presented in the module. The module was accessible online for 1 week. Only the PGY 2 residents participated in the role-play sessions, serving as an intervention group. PGY 3 residents did not participate in additional activities beyond the assigned module (control).

At the end of boot camp, an identical online assessment of confidence and knowledge regarding signs of burnout, substance abuse, and mood disorders was distributed to both PGY 2 and PGY 3 residents (Appendix C). Three months later, the same residents were assigned an additional online assessment (Appendix D) of their ability to recognize maladaptive behaviors using a standardized video (Appendix E).

### Video Scenarios and Skill Assessment

In the 3-month delayed assessment, both cohorts viewed a previously published video created by Dr. Lee Ann Riesenberg and colleagues^19^ that depicted a simulated stressed resident peer-to-peer interaction (Appendix E). The video featured a male actor portraying a resident “manifesting some maladaptive signs of stress, including being abrupt with a colleague, irritable, and having poor eye contact while communicating.”^19^ The video was independently coded by two authors for behaviors common to those covered in both the online module and role-play vignettes.

The delayed online assessment (Appendix D) tested subjects’ ability to recognize maladaptive behaviors depicted in the video. Behaviors that had been identified in the video by both raters were provided as answer choices (correct) in the postvideo assessment along with distractor behavior options not observed by either rater (incorrect). Behaviors coded by only one rater were not included.

### Data Collection and Statistical Analysis

Surveys were conducted online using Qualtrics software through an institutional license. Due to a low response rate from the PGY 3 group, comparative analysis was not performed between the cohorts for the initial premodule survey and postmodule survey. Statistical comparison of the PGY 2 cohort's premodule and postmodule responses was performed with the Fisher exact test. We categorized learners indicating *agree* or *strongly agree* on a Likert scale as confident. We categorized remaining responses as not confident. We used the Student *t* test with α < .05 as the cutoff for significance to compare PGY 2 and PGY 3 responses on the delayed assessment survey. Microsoft Excel was used for statistical analysis.

## Results

Eighteen neurology residents completed a divided educational activity on the recognition of maladaptive responses to occupational stress. Nine PGY2 residents participated in a role-play activity while nine PGY3 residents did not. Response rates on premodule surveys were low, with four of nine (44%) PGY 2 residents responding and one of nine (11%) PGY 3 residents responding. Table 1 describes the results for the PGY 2 cohort. Few PGY 2 respondents scored confident regarding their knowledge of resources provided at our institution for those struggling with burnout (zero of four), substance abuse (one of four), or mood disorders (zero of four). The majority responded as confident they were able to identify symptoms of burnout, substance abuse, and mood disorders in both themselves and in their peers.

**Table 1. t1:**
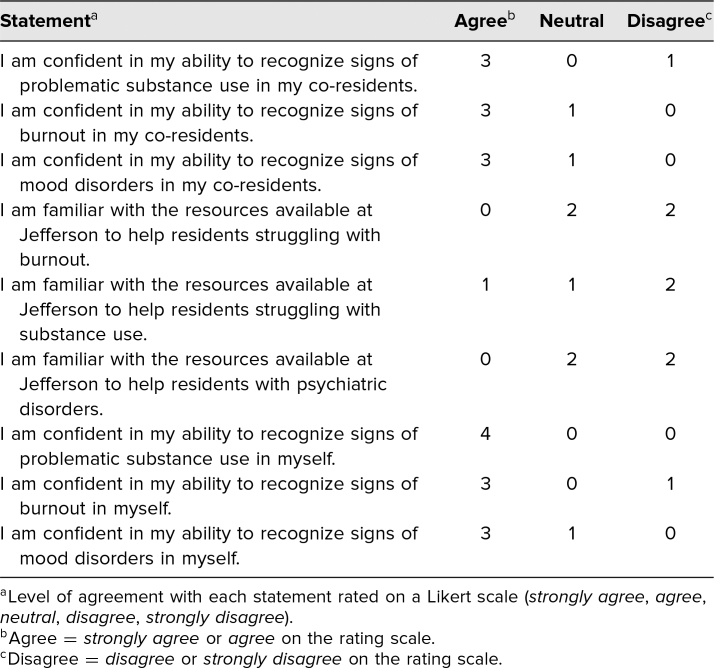
Baseline Results From the Survey Before the Online Module and Role-Play

Response rates on the postmodule assessment were also low, with four of nine (44%) PGY 2 residents responding and one of nine (11%) PGY 3 residents responding. Results from PGY 2 respondents for the postonline module survey are presented in Table 2. Compared to before the module, all four PGY 2 respondents (100%), a significant increase, expressed confidence in their knowledge of institutional resources for burnout (*p* < .01), substance abuse (*p* < .001), and mood disorders (*p* < .01), respectively. Confidence of residents in their ability to recognize symptoms of burnout, substance abuse, and mood disorders within themselves and their peers remained high.

**Table 2. t2:**
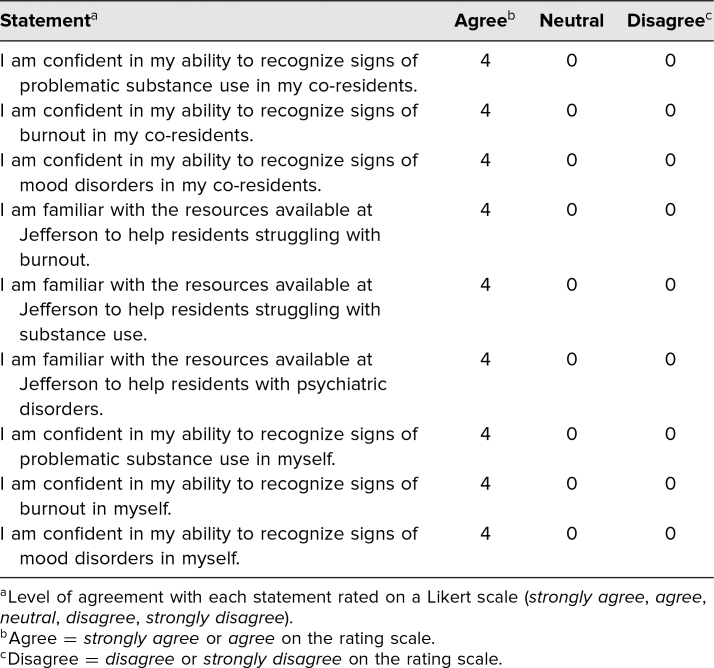
Results From the Survey After the Online Module and Role-Play

Among all[Table t1][Table t2] learners, the multiple-choice score mean was not different before (60%, *SD* = 14%) compared to after (53%, *SD* = 9%) the online module was available (*t* test, *p* = .39). Among PGY 2 residents, the multiple-choice score mean was not different before (58%, *SD* = 16%) compared to after (52%, *SD* = 10%) the completion of the two educational activities (*t* test, *p* = .55). These results are included in Table 3.

**Table 3.**
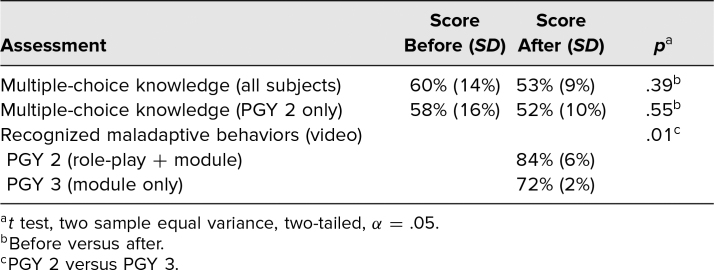
Correct Scores Before and After the Educational Activity

Resident participation in the video-based assessment was higher than in the prior surveys, with eight of nine PGY 2 participants and three of nine PGY 3 participants. In the final survey, PGY 2 residents recognized a higher proportion of maladaptive behaviors in the video (84%, *SD* = 6%) than PGY 3 subjects (72%, *SD* = 2%; *t* test, *p* = .01; Table 3).

## Discussion

We implemented a multifaceted learning activity with neurology residents to increase the recognition of behaviors indicative of maladaptive occupational stress response, such as burnout, substance use, or mood disorders. This activity used an online module to deliver semantic knowledge about the scale of burnout in medicine and among neurologists. The module also used interactive elements to help learners practice linking behaviors with categories of maladaptive stress. The module was augmented with a role-play activity that allowed residents to practice the conversations surrounding these maladaptive responses in their peers. These tools were implemented as part of a boot camp curriculum in which PGY 2 neurology residents were covering multiple topics regarding their transition to neurology training from their PGY 1 year in internal medicine. In an era in which educators of physician trainees should empower their residents with the skills to recognize maladaptive responses to occupational stress, we offer an educational tool intended to develop that skill set.

### Strengths of the Activity

The available module can be administered via a web browser and completed asynchronously by learners. While this module was completed before the COVID-19 pandemic, such online educational tools are of increased educational value in a time of mandatory social distancing. Although delivered during a specific portion of our annual educational program, this module could be offered at any time of the year or made available in any web-based learning platform for periodic review and reference by learners. The role-play activity required minimal technological or personnel support. The facilitators for the role-play were PGY 4 residents without prescribed training, which made implementation easier. Scripts were printed on paper for distribution to learners, which made technological requirements essentially nil. While not done in this scenario, we imagine that this activity could be translated to an online video conference environment, such as Zoom, with only mild adaptation needed.

The impact of the activity was positive. Learners completing the activity reported increased confidence in their recognition skills as well as confidence in the resources available at our institution. In our delayed summative assessment, the PGY 2 cohort who had participated in the role-play demonstrated better ability to identify behavioral indicators of burnout, substance abuse, and mood disorders in the video scenario compared to their PGY 3 peers who had been assigned only the online module. This suggests that the role-play improved the ability to identify such behaviors.

### Limitations of the Activity

We did not assess the activity on a lower level in the Kirkpatrick paradigm^25^; we do not know if the learners found either the module or the role-play to be enjoyable. Conversations surrounding mood disorders and substance abuse inevitably occurred during the role-play activity. We acknowledge that such simulated scenarios and the conversations surrounding them can be upsetting for many individuals, particularly those who may have been previously personally affected by them. The facilitators in this activity were senior resident peers, without formal training in facilitating these potentially triggering conversations. Our activity failed to increase multiple-choice knowledge assessment scores in an uncontrolled before-and-after analysis. However, low participation rates and thus very low sample sizes limited the ability to draw definitive conclusions from the data.

While the module was made available over the specified time period to all residents between assessments, we did not mandate or track completion of the module among residents. This limited conclusions about whether the information covered in the module was actually viewed by the subjects who completed pre- and postmodule assessments. For educators seeking to track learners’ engagement, mechanisms to track completion of the module may be available through a number of hosting platforms such as Google Analytics or Canvas.

Selection bias may be present if residents struggling from burnout or who harbor dismissive attitudes about burnout are less likely to engage with educational activities surrounding the topic itself. Our resident cohort consisted solely of neurology trainees. Thus, generalizability of both our education resources and our data may not apply to residents in other fields. While the experience of different residency specialties with regard to burnout may show differences in burnout rates,^10^ the impact of these differences on residents’ ability to recognize maladaptive behaviors has not been studied. We hope our activity could be adapted to other specialties’ training programs in an effort to increase the ability to generalize this intervention.

As stated, the intervention cohort of PGY 2 residents performed better at recognizing maladaptive behaviors than their control cohort PGY 3 peers. While our study may suggest that this was a function of the role-play activity intervention, other explanations inherent to differences in these groups do exist, specifically that they were at different stages of training. It is possible that additional years of residency training, during which residents were exposed to repeated occupational stress, decreased the ability to identify, or the motivation to identify, evident symptoms. There may be a phenomenon present where repeated exposure to the occupational stress, long work hours, low sense of job control, and poor work-life balance associated with residency training may ultimately reduce resident sensitivity to the burnout syndrome. A relationship between these formal recognition abilities and burnout among subjects has not been specifically explored and represents a potential area for future study.

The video example was a onetime assessment. A baseline assessment, using a similar but different video, could be introduced into the activity, and learners’ performance on recognizing behaviors could be assessed before and after the educational activity. By using a different video, improvement based solely on familiarity could be avoided. This may provide a better assessment of whether there is a perceptible change in the skill being taught.

### Implementing the Activity

While the content of the role-play scenarios made direct reference to inpatient neurologic services at Jefferson, the scripts can be easily edited and adapted to a multitude of residency training programs in other specialties. Portions of the script where edits can be made are formatted in boldface and enclosed within brackets. This should allow for easy adoption by other specialties’ programs.

Resident physicians suffer from high rates of burnout across specialty disciplines.^10^ The residency experience is also impaired by substance abuse and mood disorders in trainees.^13–15^ Physician suicide rates are significantly higher than the general population.^17^ As result, interventions must be implemented that expedite the intervention for residents in distress. We feel that educational activities targeting residents and their ability to recognize signs of distress in the context of their peer relationships can be an effective intervention for resident educators to implement. We provide here an educational activity that may facilitate skill-building among resident trainees in the work of recognizing signs of impairment in themselves and each other.

## Appendices

Online Learning Module folderRole-Play Activity Script.docxPre- and Immediate Postsurvey.docx3-Month Postsurvey.docxStressed Resident Interaction Video.wmv
*All appendices are peer reviewed as integral parts of the Original Publication.*
