# Supplementary material for: An Educational Workshop to Improve Neurology Resident Understanding of Burnout, Substance Abuse, and Mood Disorders
Source: MedEdPORTAL. 2021 Jul 1;17:11164. doi: 10.15766/mep_2374-8265.11164 (PMC8245593; doi:10.15766/mep_2374-8265.11164)
Supplement: Supplementary file 1 — Online Learning Module folderRole-Play Activity Script.docxPre- and Immediate Postsurvey.docx3-Month Postsurvey.docxStressed Resident Interaction Video.wmv [file mep_2374-8265.11164-s001.zip › B. Role-Play Activity Script.docx]

**Role-Play Script**

**Appendix B**

**Case 1.**

***Instructions****: This is a 15 minute exercise. Please take 2-3 minutes to read your role on your own. Then, within your three person group, you may begin your role-plays—****Resident B will initiate the conversation first.*** *After you’ve completed the encounter, the observer will lead a brief discussion. After your group has done each of the three cases then everyone will meet to discuss each small groups observations and conclusions.*

**Your role is: Resident A**

You are feeling burnt out. You are a neurology [**may substitute alternative specialty]** resident, almost done with your PGY2 year **[may substitute another year heavy on inpatient service]**, having 2 months completed of a long 3 month inpatient stretch. The amount of paperwork you have and consequently less time for patients is taxing. You are frustrated about the lack of day to day patient interaction, feeling you work “behind the scenes”. You feel like senior resident is not pulling his weight of work to do. You feel the fellow puts more scutwork on you than on the other junior resident, Resident B. You worry you sometimes don’t care about how your patients fare. More than once you’ve caught yourself referring to your patients by “diagnosis” instead of their name. You often use dark humor to cope with all this stress. Getting up every morning to come to work seems more and more exhausting. You feel like you have no time to learn and improve to become a better neurologist.

**Communication Challenge: Do not use the phrase “burn out” throughout the conversation.**

**Scenario:**

You are the current stroke **[substitute service with high workload in your program]** junior resident and you are walking with another junior resident, Resident B, back to the **hospital**  from noon conference. You both have just seen a deceased patient being wheeled out of the back of the hospital to a funeral director’s van. You just pointed to the patient and joked, “Hey look, another satisfied customer”. **Resident B is about to respond to your comment toward the deceased patient.**

**Case 1.**

***Instructions****: This is a 15 minute exercise. Please take 2-3 minutes to read your role on your own. Then, within your three person group, you may begin your role-plays—****Resident B will initiate the conversation first.*** *After you’ve completed the encounter, the observer will lead a brief discussion. After your group has done each of the three cases then everyone will meet to discuss each small groups observations and conclusions.*

**Your role is: Resident B**

You are a resident almost done with your PGY2 year. Your year has been really busy but you feel that the hard work has made you a better physician. You’ve struggled with not knowing answers the attendings’ questions about stroke and encephalitis **[insert diagnoses germane to your specialty]**, but feel you’re getting better at it. You enjoy taking care of patients and working to make unusual diagnoses. You work hard during the day so you have time to go out for happy hours with your friends in internal medicine when you can. You also started going to the gym again (mostly on the weekends but hey it’s a start!).

It’s not all been easy. You’ve taken advantage of **[your institution’s counseling center]** for counseling every other week. Residency has been stressful at times, and it’s nice to be able to talk to someone besides your spouse (who still doesn’t understand the medical world). And you have found it to be very helpful (and it’s free which is a bonus!).

Scenario:

You are a junior resident on [stroke] walking with Resident A (also junior) to the hospital from noon conference towards the end of your PGY2 year. You both have just seen a deceased patient being wheeled out of the back of the hospital to a funeral director’s van. Resident A just pointed to the patient and joked, “Hey look, another satisfied customer”. You are very uncomfortable with the joke about the deceased patient and this isn’t the first time Resident A has made a joke like this. **You respond to the comment toward the deceased patient.**

**Case 1.**

***Instructions****: This is a 15 minute exercise. Please take 2-3 minutes to read your role on your own. Then, within your three person group, you may begin your role-plays—****Resident B will initiate the conversation first.*** *After you’ve completed the encounter, the observer will lead a brief discussion. After your group has done each of the three cases then everyone will meet to discuss each small groups observations and conclusions.*

**Your role is: Observer**

Resident A (junior) and Resident B are nearing the end of their PGY2 **[Neurology]** year. Both have had stretches of busy rotations on **[Stroke, ICU, and Wards].** They were walking back from noon conference when they see a deceased patient being wheeled out of the back of the hospital into a funeral director’s van. Resident A points to the patient and jokes, “Hey, look! Another satisfied customer.”

**Talking Points** (discuss after role-play)

1. What negative experiences are adversely affecting Resident A? How would you describe their current psychological state?

**Resident A is Burnt Out. S/he has high job demand with a number of clinical tasks. The resident notes feeling disconnected from interacting with patients which possibly threatens the personal meaning found in the work being done. The insensitive joke at the end is characteristic of Depersonalization, a component of burnout where the humanistic qualities of our patients are under-appreciated.**

1. What behaviors is Resident A displaying?

**Insensitive regards towards patients; lack of empathy for patients; referring to patients as a diagnosis instead of name; exhaustion when it comes to going to work**

1. What coping mechanisms (good or bad) is Resident A displaying?

**Humor (albeit dark)**

1. Which behaviors/coping mechanisms are adaptive and which are maladaptive?

**Adaptive: reflecting on the meaning of physicians’ work, mindfulness, spending time with family; seeking professional help (counseling)**

**Maladaptive: insensitive humor; disparaging others (especially patients); blaming others**

1. What resources is Resident B using? What additional resources could both residents access? What strategies could they employ to mitigate burnout?

**Taking advantage of counseling services, making time for self-care (socializing, exercise). Both residents could work on doing these things. Other strategies to mitigate burnout include discussing the difficulties of work with each other; self-reflection; mindful meditation**

1. How can Resident B be more supportive of their colleague who is resistant to any advice? If you have a co-resident who you notice is burnt out, what strategies can you use or resources you can access to help them?

**Observe how Resident B responds to Resident A. Take some time to reflect on the interaction and discuss it as a group. Brainstorm together what your group knowns about strategies and resources you learned about in the module.**

**Case 2.**

*Instructions: This is a 15 minute exercise. Please take 2-3 minutes to read your role on your own. Then, within your three person group, you may begin your role-plays—****Resident B will initiate the conversation first.*** *After you’ve completed the encounter, the observer will lead a brief discussion. After your group has done each of the three cases then everyone will meet to discuss each small groups observations and conclusions.*

Your role is: Resident A

You feel depressed. It’s affecting your work. You have experienced an episode of major depression during medical school, but have not shared this with co-residents, even with those who you consider to be friends. Lately, you have been having trouble falling asleep for the past month, worrying about the status of your relationship. You feel immense guilt at neglecting your teaching duties at work and your family duties at home. You have been successful at suppressing these feelings for the first two years of residency, and have always been known as the resident who goes the extra yard for their patients. You are well known as the happy-go-lucky, bubbly personality that everyone loves.

You are the senior resident on the **[Consult service]**, working for the past few weeks with your junior resident, Resident B. You won a peer-teaching award last year and loved teaching students and juniors, but you don’t really feel like it anymore. Resident B has expressed frustration during this rotation about your lack of teaching. You feel terrible you were late to rounds two days this week because it was hard to get out of bed. Your attending noticed and this only made you feel more guilty. You try to steal naps in the call room because you’re so tired. You have forgotten to put in key orders on admissions, but nothing serious has come of it. Yet.

Things at home are not good either. Your spouse is worried about you and has urged you to “talk to someone”. You are reluctant to do so, fearing the possible consequences of disclosing psychiatric illness on licensure applications. Your spouse is very frustrated, feeling like you’re hard to help. This only compounds your guilt about how you’ve felt over the past few months.

**Communication Challenge: Be initially reluctant to share feelings of anhedonia or low mood. Instead focus on the more non-specific symptoms of depression at first.**

**Scenario:**

You are the senior resident on the **[neurology consult]** service, working with Resident B, the junior resident. It’s 4:25 PM **[almost end of shift]** and the ED has just called the team about a case of **[altered mental status**]. Resident B eagerly runs down to see the patient and then returns precisely at 4:55pm to present the patient to you. Halfway through the resident’s presentation, you realize you weren’t paying attention and can’t follow along. You interrupt them, saying “Hey, can you start over? I sort of zoned out there for a minute.” **Resident B is about to respond to your request.**

Case 2.

*Instructions: This is a 15 minute exercise. Please take 2-3 minutes to read your role on your own. Then, within your three person group, you may begin your role-plays—****Resident B will initiate the conversation first.*** *After you’ve completed the encounter, the observer will lead a brief discussion. After your group has done each of the three cases then everyone will meet to discuss each small groups observations and conclusions.*

**Your role is: Resident B**

You are the junior resident working with senior Resident A on the [**neurology consult]** service for the past three weeks. You were excited to work with Resident A at the beginning of the rotation, because you heard from your junior co-residents that they are amazing to work with. Resident A even won the peer-nominated award last year for their excellent patient care and enthusiasm for teaching. However, your three weeks with Resident A have not been what you expected. Resident A is frequently late to sign out in the morning, and the night team has become very frustrated with both Resident A and you. Your attending spoke to you both a few days ago, expressing his disappointment with late progress notes and unfollowed plans. You have at times felt as if Resident A has left you to make decisions in scenarios where you felt senior guidance was appropriate. Last week, you pulled Resident A aside and asked them if you were doing something wrong, since Resident A is so well-liked and well-regarded by your colleagues. Resident A assured you that you have done nothing wrong. You have been concerned about Resident A, but you are also frustrated with being paired with them during this rotation.

**Scenario:**

Resident A is the senior resident on the [**neurology consult**] service, working with you, the junior resident. It’s 4:25 PM [almost end of shift] and the ED has just called the team about a case of **[altered mental status].** You eagerly run down to see the patient and then return precisely at 4:55pm to present the patient to Resident A. Halfway through your presentation, Resident A abruptly interrupts you, saying “Hey, can you start over? I sort of zoned out there for a minute.” **The conversation begins with you asking Resident A if everything is OK.**

Case 2.

*Instructions: This is a 15 minute exercise. Please take 2-3 minutes to read your role on your own. Then, within your three person group, you may begin your role-plays—****Resident B will initiate the conversation first.*** *After you’ve completed the encounter, the observer will lead a brief discussion. After your group has done each of the three cases then everyone will meet to discuss each small groups observations and conclusions.*

**Your role is: Observer**

**Scenario:**

Resident A is the senior resident on the **[neurology consult]** service, working with Resident B, the junior resident. It’s 4:25 PM [almost end of shift] and the ED has just called the team about a case **of [altered mental status]**. Resident B eagerly runs down to see the patient and then returns precisely at 4:55pm to present the patient to Resident A. Halfway through the junior resident’s presentation, Resident A abruptly interrupts them, saying “Hey, can you start over. I sort of zoned out there for a minute.” **The conversation begins with Resident B** asking Resident A if everything is OK.

**Talking Points (discuss after role-play)**

1. What behaviors has Resident A displayed?

**Insomnia; arriving late to work; forgetfulness of clinical-tasks; sleeping at work; inattention**

1. Are the behaviors displayed by Resident A a normal reaction to a busy residency?

**No. These are concerning of a possible mood disorder.**

1. Could Resident A be suffering from a mood disorder? What behaviors did you hear about that support this possibility?

**Yes. The resident has sleep changes, inattention, guilt, anhedonia which could call indicate depression. Please note, the role-playing resident may not reveal *all* of this.**

1. What factors likely contribute to Resident A’s reluctance to seek help?

**Stigma against mental illness in physicians; Apathy; Shame; Being under informed about what resources are available for residents**

1. What symptoms differentiate a mood disorder from burnout?

**Burnout = Occupational stress response related to work. Depression is pervasive in life at and away from work.**

1. What resources are available to Resident A? How would you approach a co-resident with concerning symptoms like Resident A’s?

**These may vary by your institution. Local counseling services and medical care is likely available. All US states have Physician Health Programs which can be utilized as well.**

**Approaching your co-residents from a place of concern (and not shame or anger) is best. If you feel uncomfortable but are concerned, speak with your chief residents or Program Director.**

1. What are some of the barriers to getting help for mental illness? How can we overcome those barriers?

**Time off from work; Stigma against mental illness; Variable insurance coverage for mental health services are some examples. All have different strategies to overcome at different levels.**

1. If you were Resident B, how would you approach checking in on your senior resident? Would you behave differently if they were your co-PGY2? What about in the future, if you see these behaviors in a junior resident?

**See #6**

**Case 3.**

*Instructions: This is a 15 minute exercise. Please take 2-3 minutes to read your role on your own. Then, within your three person group, you may begin your role-plays—****Resident B will initiate the conversation first.*** *After you’ve completed the encounter, the observer will lead a brief discussion. After your group has done each of the three cases then everyone will meet to discuss each small groups observations and conclusions.*

**Your role is: Resident A**

You think everything is going OK. You’ve managed to help take the edge off after work with a few beers. Smoking a little pot helps you get to sleep some nights. You are a PGY4 **[final year of training]** who thinks these last few months of residency are useless. You know what you’re doing and you don’t need to be here. You won an award for clinical excellence as a PGY2 and matched in fellowship months ago. Faculty evaluations have commented that you seem unenthusiastic about work but you don’t care. You know that you can’t be fired since “they need the bodies.” You’ve overheard some of your co-residents, who you used to be friendly with, have begun to express frustration when they see that they are scheduled to work with you. At last Friday’s resident happy hour, one of your friends even suggested that you cut back on your drinking, knowing you had to go in for a Saturday shift, but you feel as if they were just trying to put a damper on the party. You were feeling pretty rough the next morning, and showed up 2 hours after the fellow had to call you to see where you were. Sometimes if you’re feeling too hungover, you’ll “call out sick” and have one of your co-residents cover your shift. You can always pay them back later...

You have a family history of alcoholism, which you don’t like to discuss. You tried abstaining from alcohol in the past but it felt awkward at resident functions where everyone seems to be drinking. You’re never the only one drunk so it feels fine. You tend to drink more frequently when you are feeling stressed and overwhelmed by residency duties. You don’t see this as a problem, and assume that your fellow residents also do the same. You see all of your co-residents drink heavily at resident functions, and that you never would jeopardize patient care with your behavior.

**Communication Challenge: You suspect others in the program drink as much as you do, but you’ve not discussed it. Regardless, you’re not eager to quantify how much you drink or smoke.**

**Scenario:**

You are a senior resident on **[wards service],** and Resident B is a PGY2 on their first ICU rotation. It’s 8AM and didactic conference has begun. You are whispering with your fellow PGY4s because you don’t really care about the conference. Resident B asks you several times to quiet down but you don’t think you are being very loud. Once the conference ends, Resident B pulls you aside and asks to speak privately. You both enter one of the empty offices near the conference room, and Resident B begins. **Resident B is the first to speak.**

Case 3.

*Instructions: This is a 15 minute exercise. Please take 2-3 minutes to read your role on your own. Then, within your three person group, you may begin your role-plays—****Resident B will initiate the conversation first.*** *After you’ve completed the encounter, the observer will lead a brief discussion. After your group has done each of the three cases then everyone will meet to discuss each small groups observations and conclusions.*

**Your role is: Resident B**

You are a PGY2 currently on your first ICU rotation. Things are extra busy with the Fellow at a Critical Care conference and the NP schedule got mixed up, so the NP isn’t coming in. The attending is really particular and is not afraid to let you know when they are upset that you did not do something to their high, unrealistic expectations. You feel like everyone else has it together except for you. You were hoping to have a good PGY4 senior on with you, but were disappointed when it ended up being Resident A.

Resident A tends to drink more heavily than other residents at social gatherings and will occasionally show up to work appearing exhausted and appearing disheveled. When they are actually on time, they have even bragged about coming to work still a “little buzzed”. You initially looked up to Resident A when you started your PGY2 year, because they appeared like they had it all together. However, over the past few months, you are concerned that Resident A is jeopardizing their career with their behavior. You’ve experienced this concerning behavior yourself, when you received a very bare-bones sign out from Resident A on your last rotation, and one of your patients would have experienced an adverse event had you not caught Resident A’s error. You’ve heard from other residents that Resident A has been late to rounds frequently last month, and has called out a few times. You are worried about Resident A.

**Scenario:**

Resident A is the senior resident on **[wards service]**, and you are a PGY-2 on your first ICU rotation. It’s 8AM and didactic conference has begun. Resident A is talking with their co-PGY4s loudly and despite you asking them to quiet down multiple times, they continue to distract you from the conference. Given your concerns about Resident A and upon hearing at one point that Resident A was bragging about staying out late at the bar the night before, you decide it’s time to at least check in to make sure things are okay. Once the conference ends, you pull Resident A aside and asks to speak privately. You enter one of the empty offices near the conference room, and you attempt to explore Resident A’s struggles. **You are the first to speak.**

Case 3.

*Instructions: This is a 15-minute exercise. Please take 2-3 minutes to read your role on your own. Then, within your three-person group, you may begin your role-plays****—Resident B will initiate the conversation first.*** *After you’ve completed the encounter, the observer will lead a brief discussion. After your group has done each of the three cases then everyone will meet to discuss each small groups observations and conclusions.*

**Your role is: Observer**

**Scenario:**

Resident A is a senior resident on **[wards** **service**], and Resident B is a PGY2 on their first ICU rotation. It’s 8AM and didactic conference has begun. Resident A is talking with their peers loudly during lecture despite Resident B asking them to quiet down. Once the conference ends, Resident B pulls Resident A aside and asks to speak privately. They enter one of the empty offices near the conference room.

Talking Points (discuss after role-play)

1. What behaviors has Resident B noticed/heard about in Resident A?

**Inattention to clinical duties; Excessive alcohol intake; Working while intoxicated**

1. Are the behaviors in Resident A normal responses to residency stress?

**These are *maladaptive* responses and their substance use is having an impact on their function**

1. Could Resident A be suffering from a substance use disorder? What are the behaviors that support this?

**Yes. Their substance use is interfering with their ability to do their job and function normally. They have difficulty cutting back despite attempts. See behaviors above.**

1. Could Resident A have a mood disorder? What are the behaviors that support this?

**It is possible. There is apathy towards their job performance.**

1. Could Resident A be burnt out? What are the behaviors that support this?

**Possibly. Indifference towards their medical career may be seen in burnout. However, this emotional state is having a negative impact on their function outside of work and thus likely is more than simple burnout.**

1. What factors likely contribute to Resident A’s reluctance to seek help?

**Fear of the implications of their drinking, especially considering what they know of their family history. Revealing it may jeopardize their career.**

1. How would you approach a co-resident who demonstrated similar behaviors? What if they were a close friend? Or in a superior position?

**Approaching your co-residents from a place of concern (and not shame or anger) is best. If you feel uncomfortable but are concerned, speak with your chief residents or Program Director.**

1. What resources are available to Resident A?

**Local resources at your institution may vary so check with your PD or GME office. Alcoholics/Narcotics Anonymous is available. All US states have Physician Health Programs which can be utilized as well.**
