# Supplementary material for: An Educational Workshop to Improve Neurology Resident Understanding of Burnout, Substance Abuse, and Mood Disorders
Source: MedEdPORTAL. 2021 Jul 1;17:11164. doi: 10.15766/mep_2374-8265.11164 (PMC8245593; doi:10.15766/mep_2374-8265.11164)
Supplement: Supplementary file 1 — Online Learning Module folderRole-Play Activity Script.docxPre- and Immediate Postsurvey.docx3-Month Postsurvey.docxStressed Resident Interaction Video.wmv [file mep_2374-8265.11164-s001.zip › C. Pre- and Immediate Postsurvey.docx]

Pre- and Post-Activity Assessment
(Appendix C)

[Correct Answers in Bold]

# 1. Please specify your year of neurology training

PGY2

PGY3

2. Please rate your agreement with the following statements:

I am confident in my ability to recognize signs of problematic substance use in my co-residents.

I am confident in my ability to recognize signs of burnout in my co-residents

I am confident in my ability to recognize signs of mood disorders in my co- residents.

I am familiar with the resources available at [my institution] to help residents struggling with burnout.

I am familiar with the resources available at [my institution] to help residents struggling with substance use.

Strongly disagree

Somewhat disagree

Neither agree nor disagree

Somewhat agree

Strongly agree

I am familiar with the resources available at [my institution] to help residents with psychiatric disorders.

I am confident in my ability to recognize signs of problematic substance use in myself.

I am confident in my ability to recognize signs of burnout in myself.

I am confident in my ability to recognize signs of mood disorders in myself.

Strongly disagree

Somewhat disagree

Neither agree nor disagree

Somewhat agree

Strongly agree

3. Which features below help define substance dependence beyond substance abuse? (Check all that apply)

**A. Development of tolerance to a substance** **Experiencing withdrawal symptoms from a substance**

B. Failing to meet family-related obligations due to substance use C. Failing to meet work-related obligations due to substance use D. **Using a substance in larger amounts than intended**

4. The percentage of residents who report daily use of alcohol is closest to which of the following?

A. 1%

**B. 5%**

C. 10%

D. 20%

E. 25%

5. Physician impairment is the inability of a licensee to practice medicine with reasonable skill/safety as a result of which of the following? (Check all that apply)

A. Ongoing malpractice investigation

B. Lack of empathy

**C. Mental disorder**

**D. Physical illness or condition E. Substance related disorders**

6. Which of the following are true about resident physician substance use? (Check all that apply)

**A. 1 in 2 residents report at least one episode of binge drinking in prior year**

B. Increasing training year in resident classes (eg. PGY3 vs. PGY2) correlates with higher binge drinking rate

**C. 1 in 6 residents report binge drinking once per month**

**D. Resident physicians demonstrate higher rates of alcohol and benzodiazepine use than age matched peers**

E. Surveyed attending neurologists had the highest percentage (10%) of respondents who scored positive for alcohol abuse among medical specialties surveyed.

7. Which of following behaviors in your colleague may raise suspicion for problematic substance use? (Check all that apply)

**A. Failing to show up for a shift B. Injected conjunctiva**

C. Frequent complaints about call schedules

D. Lacking empathy towards ill patients

**E. New financial difficulties**

8. What resources do residents have for substance abuse? (Check all that apply)

**A. Alcoholics/Narcotics Anonymous**

**B. [Local Institutional Counseling Center]**

**C. International Doctors in Alcoholics Anonymous**

D. [Local City/Town] Student Personal Counseling Center

**E. [State] Physician Health Program**

9. Per a 2018 JAMA Survey following medical students into residency, approximately what percentage of PGY2 resident physicians reported symptoms of burnout?

A. 15%

B. 25%

C. 35%

**D. 45%**

E. 55%

10. Which of the following is/are true about resident physician burnout? (Check all that apply)

A. Finding meaning in clinical makes physicians prone to burnout.

**B Job demands impeding work/personal life balance make physicians prone to burnout**

**C. Personal factors affecting the ability to buffer the effect of stress make physicians prone to burnout.**

D. Higher measures of empathy as a medical student make residents more likely to report burnout.

E. Being male is associated with higher reported symptoms of burnout during residency.

11. Which of following behaviors in your colleague may raise suspicion for mood disorder? (Check all that apply)

**A. Insomnia**

**B. Impaired concentration**

C. Unsteady gait

D. Frequently organizing group happy hours

E. **Expressing guilt about their job performance**

12. Which option is the best way to distinguish depression from burnout?

A. Increase in appetite is seen in burnout, while decrease in appetite is seen in depression.

B. Depression must be present for 2 weeks while burnout does not.

**C. Depression symptoms appear outside of work, while burnout symptoms are work related.**

D. There is no good way of distinguishing between depression and burnout as they are too similar.

E. Depression is rare among physicians while burnout is common.

13. Which of following behaviors in your colleague may raise suspicion for physician burnout? (Check all that apply)

**A. Irritability towards co-workers**

B. Impaired coordination

**C. Referring to patients by their diagnosis**

**D. Self-deprecating comments about job performance**

E. Passive ideation about their own death
