# Supplementary material for: An Educational Workshop to Improve Neurology Resident Understanding of Burnout, Substance Abuse, and Mood Disorders
Source: MedEdPORTAL. 2021 Jul 1;17:11164. doi: 10.15766/mep_2374-8265.11164 (PMC8245593; doi:10.15766/mep_2374-8265.11164)
Supplement: Supplementary file 1 — Online Learning Module folderRole-Play Activity Script.docxPre- and Immediate Postsurvey.docx3-Month Postsurvey.docxStressed Resident Interaction Video.wmv [file mep_2374-8265.11164-s001.zip › D. 3-Month Postsurvey.docx]

Impairment Delayed Post Test (Appendix D)

Start of Block: Default Question Block

Q1 What is your year of residency training?

- PGY-2
- PGY-3

Q2 *[The embedded video was hosted on YouTube. It is available as Appendix F. See citations below^1^]*

Please observe the following interaction between two residents.

| 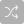 |
| --- |

Q3 Which of the following characteristics/behaviors are exhibited by the resident sitting at the computer in the video?

- Jeopardizes patient safety due to substance use
- **Irritability**
- **Isolation from peers**
- Appropriate use of substances
- **Missing out on family obligations**
- **Low sense of personal achievement**
- Appropriate use of humor
- Suppression of stressors
- Failing to meet work obligations due to substance use
- **Emotional Exhaustion**
- Suicidality
- Displays altruism
- **Job strain**
- **Pessimism**
- Periods of hyperactivity
- **Cynicism**
- **Fatigue**
- Appropriate work life balance
- **Decreased Energy**
- Low mood

| 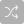 |
| --- |

Q4 Which features of the residents' working environment may contribute to burnout, substance abuse, or mood disorders?

- **Low job control**
- **High Job Strain**
- Low Job Strain
- **High job demand**
- Low job demand
- High job control
- **Isolation from peers**
- **Isolation from family**
- Support from peers
- **Long work hours**
- **Demanding attendings, ancillary staff, and patients**

End of Block: Default Question Block

Start of Block: Block 1

Q5 The resident on the clip is under a great deal of stress.

- Strongly disagree
- Somewhat disagree
- Neither agree nor disagree
- Somewhat agree
- Strongly agree

Q6 The resident on the clip is impaired.

- Strongly disagree
- Somewhat disagree
- Neither agree nor disagree
- Somewhat agree
- Strongly agree

Q7 The resident on the clip needs to seek professional counseling.

- Strongly disagree
- Somewhat disagree
- Neither agree nor disagree
- Somewhat agree
- Strongly agree

Q8 The problems the resident has are primarily related to at home issues.

- Strongly disagree
- Somewhat disagree
- Neither agree nor disagree
- Somewhat agree
- Strongly agree

Q9 The resident in the clip should be removed from the service.

- Strongly disagree
- Somewhat disagree
- Neither agree nor disagree
- Somewhat agree
- Strongly agree

Q10 The resident on the clip is not receiving adequate support from the residency program.

- Strongly disagree
- Somewhat disagree
- Neither agree nor disagree
- Somewhat agree
- Strongly agree

Q11 The resident on the clip is not receiving adequate support from the attending physician.

- Strongly disagree
- Somewhat disagree
- Neither agree nor disagree
- Somewhat agree
- Strongly agree

Q12 The resident on the clip has a substance abuse problem.

- Strongly disagree
- Somewhat disagree
- Neither agree nor disagree
- Somewhat agree
- Strongly agree

Q13 The resident on the clip is a risk to patient safety.

- Strongly disagree
- Somewhat disagree
- Neither agree nor disagree
- Somewhat agree
- Strongly agree

Q14 The resident on this clip is a good role model.

- Strongly disagree
- Somewhat disagree
- Neither agree nor disagree
- Somewhat agree
- Strongly agree

Q15 The resident on this clip demonstrates many of the common stressors that residents face today.

- Strongly disagree
- Somewhat disagree
- Neither agree nor disagree
- Somewhat agree
- Strongly agree

Q16 The resident on this clip should be able to resolve these issues her/himself.

- Strongly disagree
- Somewhat disagree
- Neither agree nor disagree
- Somewhat agree
- Strongly agree

End of Block: Block 1

Citations:

1. Video has been used with permission from Dr. K. Berg. It is adapted from: Berg K, et al. Early Detection and Intervention for the Stressed Resident, 5/21/2013.

DOI: <https://doi.org/10.15766/mep_2374-8265.9419>.

It’s used under a Creative Commons license: Attribution-NonCommercial-NoDerivatives 4.0 International (CC BY-NC-ND 4.0)
