# Supplementary figures and images for: An Educational Workshop to Improve Neurology Resident Understanding of Burnout, Substance Abuse, and Mood Disorders
Source: MedEdPORTAL. 2021 Jul 1;17:11164. doi: 10.15766/mep_2374-8265.11164 (PMC8245593; doi:10.15766/mep_2374-8265.11164)

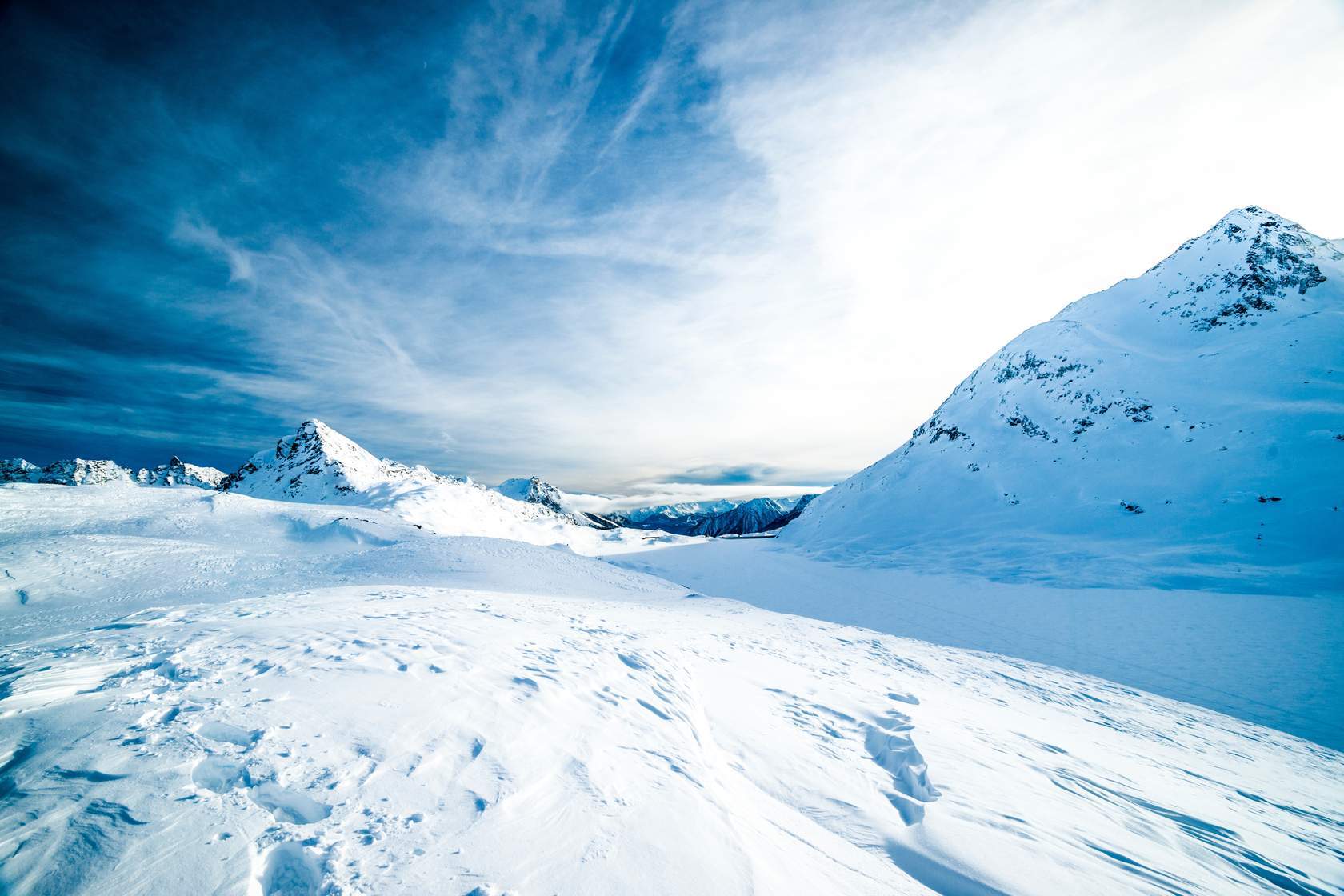

Supplement: Supplementary file 1 — Online Learning Module folderRole-Play Activity Script.docxPre- and Immediate Postsurvey.docx3-Month Postsurvey.docxStressed Resident Interaction Video.wmv [file mep_2374-8265.11164-s001.zip › A. Online Learning Module/content/assets/DIp9QNYX0GCSF5EW_mountains.jpg]

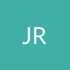

Supplement: Supplementary file 1 — Online Learning Module folderRole-Play Activity Script.docxPre- and Immediate Postsurvey.docx3-Month Postsurvey.docxStressed Resident Interaction Video.wmv [file mep_2374-8265.11164-s001.zip › A. Online Learning Module/content/assets/jn36qGzILjXIBcQA_small.png]
